# Correction: Taste of time: A porous-medium model for human tongue surface with implications for early taste perception

**DOI:** 10.1371/journal.pcbi.1009671

**Published:** 2021-12-07

**Authors:** Zhenxing Wu, Kai Zhao

In Eq 1 in the Methods, there is an error in the equation. Please view the complete, correct equation here:

$$\varepsilon =1-\frac{V_{s}}{V_{t}}$$ Eq 1

## References

[1] Wu Z, Zhao K (2020) Taste of time: A porous-medium model for human tongue surface with implications for early taste perception. PLoS Comput Biol 16(6): e1007888. 10.1371/journal.pcbi.1007888 32497080 PMC7271999

