# Supplementary material for: Taste of time: A porous-medium model for human tongue surface with implications for early taste perception
Source: PLoS Comput Biol. 2020 Jun 4;16(6):e1007888. doi: 10.1371/journal.pcbi.1007888 (PMC7271999; doi:10.1371/journal.pcbi.1007888)
Supplement: S1 Fig — (A) Case 1: Kp (permeability) = 1.18×10^−9^ m2, ε (porosity) = 0.63; (B) Case 2: Kp = 2.78×10^−9^ m2, ε = 0.79; (C) Case 3: Kp = 7.05×10^−9^ m2, ε = 0.92. Parameters for each case are listed in Table 1. (DOCX) [file pcbi.1007888.s001.docx]

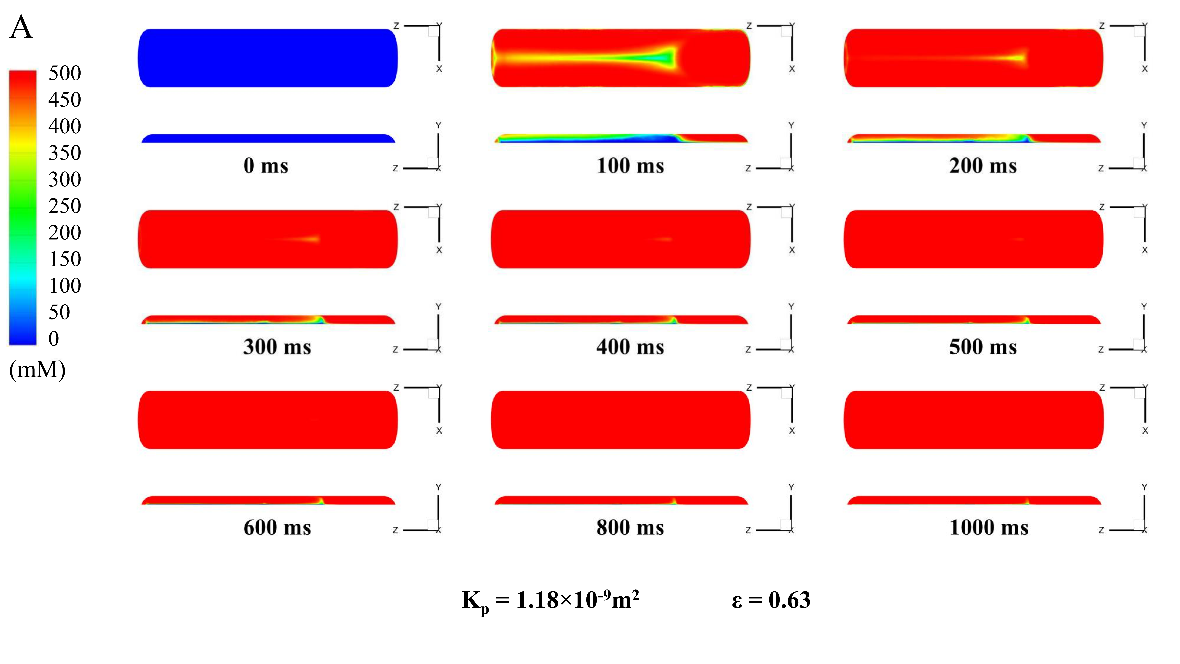


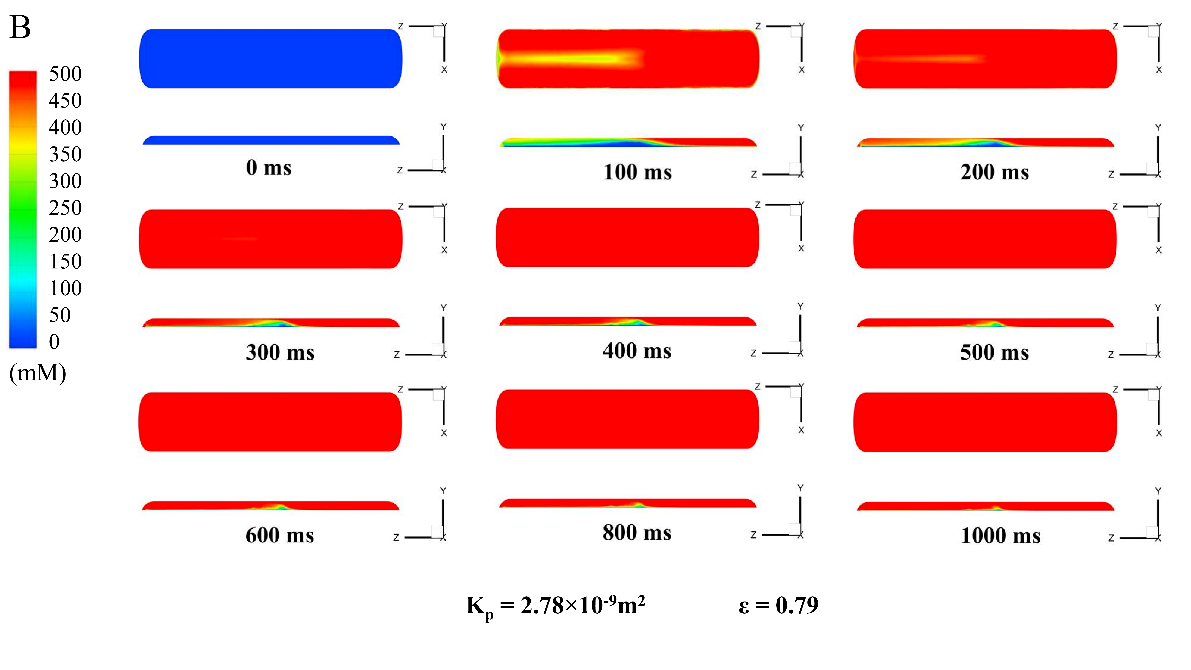


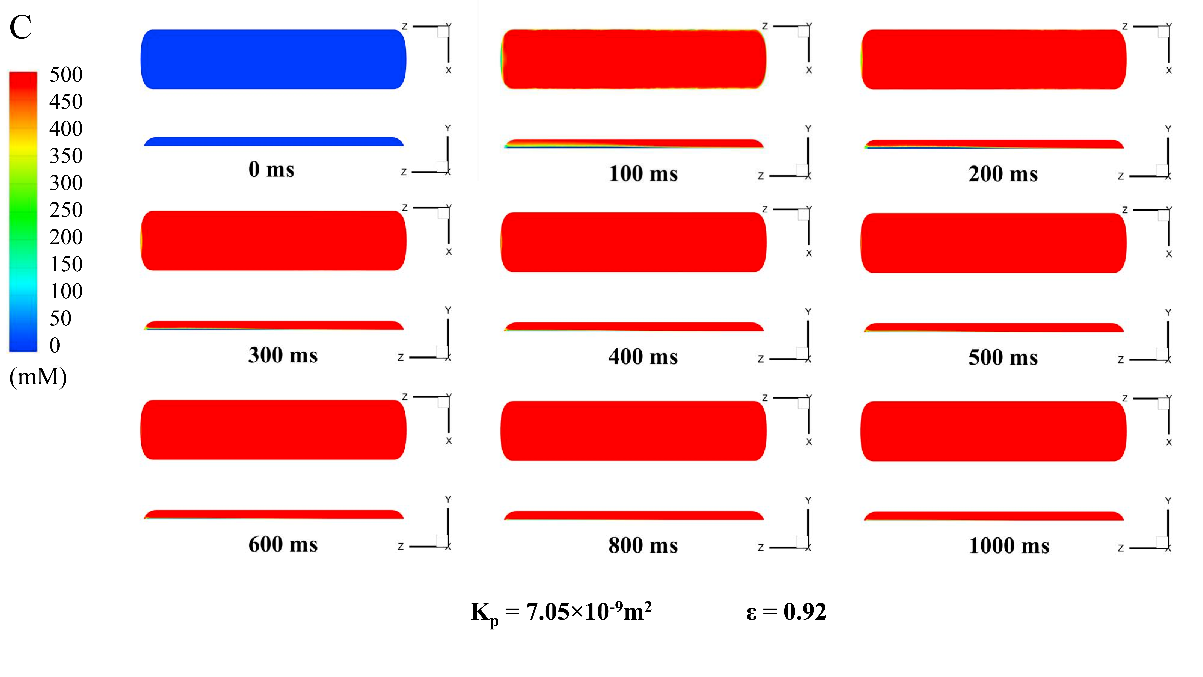


**S1 Fig.** **Top-surface view (top row) and cross-section view (bottom) of simulated NaCl concentration profile throughout the papilla layer at different time points, with stimulus of 500mM NaCl. (A)** Case 1: K_p_ (permeability)=1.18×10^-9^ m^2^, ε (porosity)=0.63; (**B)** Case 2: K_p_=2.78×10^-9^ m^2^, ε=0.79; **(C)** Case 3: K_p_=7.05×10^-9^ m^2^, ε=0.92. Parameters for each case are listed in Table 1.
