# Supplementary material for: Taste of time: A porous-medium model for human tongue surface with implications for early taste perception
Source: PLoS Comput Biol. 2020 Jun 4;16(6):e1007888. doi: 10.1371/journal.pcbi.1007888 (PMC7271999; doi:10.1371/journal.pcbi.1007888)
Supplement: S1 Table — (DOCX) [file pcbi.1007888.s002.docx]

**S1 Table. Intensity perception ratings and simulated stimulus concentrations for different pulse durations of NaCl and NaSac stimuli**

| **Measure** | **500mM NaCl solution** | | | | **2mM NaSac solution** | | | |
| --- | --- | --- | --- | --- | --- | --- | --- | --- |
| Intended pulse duration (ms) | 100 | 200 | 300 | 1000 | 100 | 200 | 300 | 1000 |
| Intensity rating^*^ | 11±0.1 | 14±0.7 | 12±0.9 | 18±1.0 | 8±1.8 | 12.5±1.5 | 10.5±1.2 | 16±1.4 |
| Simulated tongue concentration (mM) ^†^ | 211.81 | 416.61 | 460.05 | 494.48 | 0.85 | 1.67 | 1.84 | 1.98 |

^*^Data were from Kelling and Halpern’s intensity rating experiment[1]. A 2s stimulus pulse was assigned a modulus of 20 and used as a standard against which subjects would judge in proportion of the intensity of the experimental stimulus.

^†^The correlation between the simulated tongue concentration and the experimental intensity ratings for NaCl and NaSac stimulus pulses were 0.70 and 0.82, respectively. (Also shown below)

**
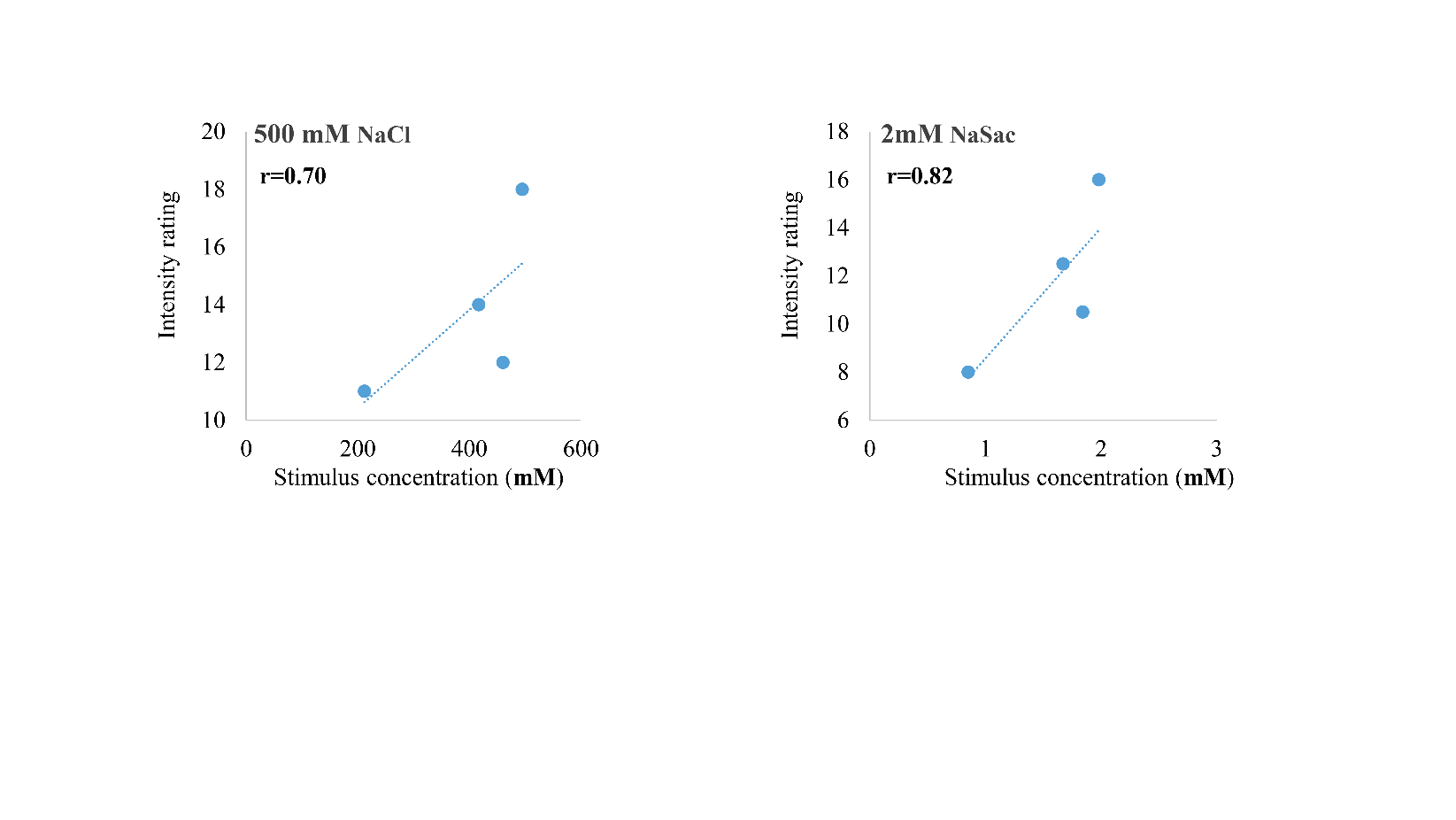
**

**References**

1. Kelling S, Halpern B. Taste flashes: reaction times, intensity, and quality. Science. 1983;219: 412. doi:10.1126/science.6849142
