# Supplementary material for: Taste of time: A porous-medium model for human tongue surface with implications for early taste perception
Source: PLoS Comput Biol. 2020 Jun 4;16(6):e1007888. doi: 10.1371/journal.pcbi.1007888 (PMC7271999; doi:10.1371/journal.pcbi.1007888)
Supplement: S2 Table — (DOCX) [file pcbi.1007888.s003.docx]

**S2 Table. Peaks of intensity perceptions and simulated peak stimulus concentrations in the tongue surface zone for various setups**

| Measure | Sucrose solution^*^ | | | | NaCl solution^†^ | | | | | |
| --- | --- | --- | --- | --- | --- | --- | --- | --- | --- | --- |
| Stimulus solution concentration (mM) | 100 | 180 | 320 | 560 | 150 | 300 | 450 | 150 | 300 | 450 |
| Holding duration (s) | 10 | 10 | 10 | 10 | 5 | 5 | 5 | 10 | 10 | 10 |
| Normalized Peak intensity rating | 2.1 | 3.5 | 6.1 | 6.9 | 20.2 | 30.0 | 41.8 | 22.5 | 35.8 | 51.7 |
| Simulated tongue concentration (mM)^‡^ | 20.7 | 37.3 | 66.2 | 115.9 | 38.8 | 77.6 | 116.5 | 54.8 | 109.5 | 164.3 |

^*^The sucrose solution intensity ratings were abstracted from the median ration judgements of taste intensity test by Lawless and Skinner[1]. The subjects in the intensity rating test were taught to move a pointer which could mark off a distance from zero proportional to the taste intensity. They were told to estimate ratios, so that if the taste intensity doubled from some level, the pointer would be moved twice as far from zero. Similarly, the subjects were instructed to preserve ratio properties between stimuli, such that if the second stimulus was half as strong at its peak as the first, the pointer would be moved half as far from zero.

^†^The NaCl solution intensity ratings were from Matuszewska et al.’s work[2]. Two reference standards were evaluated (distilled water and 2.5% NaCl solution) before each experiment in order to determine the range of intensity scale, which is from ‘none, 0’ to ‘very intensive, 100’.

^‡^The correlation between the simulated tongue concentration and the experimental peak intensity ratings for sucrose solution and NaCl solution were 0.933 and 0.993, respectively.

**Reference**

1. Lawless HT, Skinner EZ. The duration and perceived intensity of sucrose taste. Percept Psychophys. 1979;25: 180–184. doi:10.3758/BF03202983

2. Matuszewska I, Baryłko-Pikielna N. The effect of sample exposure time on the time intensity response to NaCl solutions. Food Qual Prefer. 1995;6: 43–48. doi:10.1016/0950-3293(94)P4211-N
