# Supplementary material for: Taste of time: A porous-medium model for human tongue surface with implications for early taste perception
Source: PLoS Comput Biol. 2020 Jun 4;16(6):e1007888. doi: 10.1371/journal.pcbi.1007888 (PMC7271999; doi:10.1371/journal.pcbi.1007888)
Supplement: S3 Table — (DOCX) [file pcbi.1007888.s004.docx]

**S3 Table.** **Estimated diffusivity of selected sweeteners and mean time-intensity parameters**

| Measure | Saccharin | Sucrose | Aspartame | Acesulfame K | Cyclamate | Sucralose | Crystalline  fructose |
| --- | --- | --- | --- | --- | --- | --- | --- |
| Diffusivity (×10^–10^ m^2^/s)^*^ | 7.06 | 4.76 | 4.64 | 7.73 | 6.62 | 4.55 | 7.00 |
| T_max_ (s)^†^ | 2.72 | 3.22 | 3.02 | 2.15 | 2.97 | 2.88 | 2.95 |
| M_abs_^‡^ | 10.46 | 10.39 | 7.11 | 12.95 | 11.74 | 9.72 | 11.66 |
| T_lag_(s)^§^ | 0.78 | 0.63 | 0.92 | 0.55 | 0.72 | 0.82 | 0.72 |

^*^All the diffusivities were estimated using Wilke and Chang equation[1].

^†^Time to reach peak perceived intensity[2]. The correlation coefficient between T_max_ and diffusivity is –0.689 (*p* = 0.087).

^‡^Slope to reach peak intensity[2]. The correlation coefficient between M_abs_ and diffusivity is 0.789 (*p* = 0.035).

^§^Lag time between ingestion and onset of response.[2] The correlation coefficient between T_lag_ and diffusivity is -0.545 (*p* = 0.206).

**Reference**

1. Wilke CR, Chang P. Correlation of diffusion coefficients in dilute solutions. AIChE J. 1955;1: 264–270. doi:10.1002/aic.690010222

2. Ketelsen SM, Keay CL, Wiet SG. Time-Intensity Parameters of Selected Carbohydrate and High Potency Sweeteners. J Food Sci. 1993;58: 1418–1421. doi:10.1111/j.1365-2621.1993.tb06196.x
