# Supplementary material for: Taste of time: A porous-medium model for human tongue surface with implications for early taste perception
Source: PLoS Comput Biol. 2020 Jun 4;16(6):e1007888. doi: 10.1371/journal.pcbi.1007888 (PMC7271999; doi:10.1371/journal.pcbi.1007888)
Supplement: Attachment — Submitted filename: PCOMPBIOL-D-20-00270.pdf [file pcbi.1007888.s006.pdf]

Manuscript PCOMPBIOL-D-20-00270

Dear Authors,

The manuscript "Taste of time: a porous-medium model for human tongue surface with implications for early taste perception" is interesting and with new data. Please find below my comments in details:

1. The aim of this study was clearly defined.

2. The claims of this study are partially novel.

3. The claims are properly placed in the context of the previous literature.

4. The results of this study are well described. The significance of the results of Yours study can have the influence on the future physiological research of the sense of taste.

5. The methods which were used in this study are correct. Authors attached the additional file for support of this study.

6. The manuscript contains the original data. Some of the parts of figures are supporting from the reference list.

7. The details of the methodology are sufficient to allow the experiment to be reproduced.

8. The manuscript is well organized.

9. When I was checking this manuscript I found the previously version of this manuscript on-line:

https://www.biorxiv.org/content/10.1101/780429v3

10. The nomenclature used in this manuscript is correct.
