# Supplementary material for: Taste of time: A porous-medium model for human tongue surface with implications for early taste perception
Source: PLoS Comput Biol. 2020 Jun 4;16(6):e1007888. doi: 10.1371/journal.pcbi.1007888 (PMC7271999; doi:10.1371/journal.pcbi.1007888)
Supplement: Attachment — Submitted filename: Reply letter.doc [file pcbi.1007888.s007.doc]

**Manuscript ID: PCOMPBIOL-D-20-00270.R1**

**Re: Taste of time: a porous-medium model for human tongue surface with implications for early taste perception**

Authors: Zhenxing Wu, Kai Zhao.

Dear Editors and Reviewers,

We wish to express our sincere appreciation to the reviewer for his/her insightful comments, which assisted us in substantially improving our paper. The comments raised by the reviewer have been incorporated into the revised paper with green highlight. Detailed responses are in the following.

**Reply to Reviewer # 1:**

*Reviewer #1 comment: The paper from Wu and Zhao provides an original paper on modeling the surface of the human tongue as a porous medium in order to investigate temporal taste profile. The work is highly innovative and covers a topic that is not enoght studied. It is well written and worth to be published.
I have just a minor issue that would need to be addressed. The authors discuss about taste in general but the model was applied only to NaCl, NaSac and other sweet compounds, that do not test any bitter, sour, umami compounds and do not cover all basic taste modalities, i.e. sweet, bitter, salty, sour, umami. I would suggest to re-write the title and re-phrase discussion and conclusion specifying that and softening conclusions about taste in general. Since 1983-86, publication years for the papers of Kelling and Halpern, much more is known about taste compounds and their complicated chemistry. So I think that it is important to frame the applicability of the model developed in this work to the compounds that were actually tested and discuss the possible applicability of this model for other compounds and all taste modalities as a perspective.*

Reply: This is a very good comment, we completely agree with the reviewer. We revised the abstract, addressed the compounds we simulated in this manuscript and highlighted this information in the revised manuscript in line 49, 54, 98, 207, 225, 248.

**Reply to Reviewer # 2:**

*Reviewer #2: Dear Authors,*

*The manuscript "Taste of time: a porous-medium model for human tongue surface with implications for early taste perception" is interesting and with new data. Please find below my comments in details:
1. The aim of this study was clearly defined.
2. The claims of this study are partially novel.
3. The claims are properly placed in the context of the previous literature.
4. The results of this study are well described. The significance of the results of Yours study can have the influence on the future physiological research of the sense of taste.
5. The methods which were used in this study are correct. Authors attached the additional file for support of this study.
6. The manuscript contains the original data. Some of the parts of figures are supporting from the reference list.
7. The details of the methodology are sufficient to allow the experiment to be reproduced.
8. The manuscript is well organized.
9. When I was checking this manuscript I found the previously version of this manuscript on-line:*
[*https://www.biorxiv.org/content/10.1101/780429v3*](https://mcas-proxyweb.us3.cas.ms/certificate-checker?login=false&originalUrl=https%3A%2F%2Furldefense.com.us3.cas.ms%2Fv3%2F__https%3A%2F%2Fwww.biorxiv.org%2Fcontent%2F10.1101%2F780429v3__%3B!!KGKeukY!gjx4virWWIxzSdVSmksu2bwMnjcR3AqJCmUnpXcQ3yy0lNRgqwi0HSMtHtKGLlLTLj5q$) *10. The nomenclature used in this manuscript is correct.*

Reply: Thanks for reviewer’s kindly comments.
